# Advance care planning for patients with end-stage kidney disease on dialysis: narrative review of the current evidence, and future considerations

**DOI:** 10.1007/s40620-023-01841-3

**Published:** 2024-01-18

**Authors:** S. F. Adenwalla, P. O’Halloran, C. Faull, F. E. M. Murtagh, M. P. M. Graham-Brown

**Affiliations:** 1 Department of Cardiovascular Sciences, University of Leicester, Leicester, LE1 9HN UK; 2 NIHR Leicester Biomedical Research Centre, Glenfield Hospital, Leicester, UK; 3 John Walls Renal Unit, University Hospitals Leicester NHS Trust, Leicester, UK; 4 School of Nursing and Midwifery, Medical Biology Centre, Queen’s University Belfast, Belfast, UK; 5 Leicestershire and Rutland Organisation for the Relief of Suffering (LOROS) Hospice, Leicester, UK; 6 Wolfson Palliative Care Research Centre, Hull York Medical School, University of Hull, Hull, UK

**Keywords:** Advance care planning, Dialysis, End-stage kidney disease, End of life, Palliative

## Abstract

Patients with end-stage kidney disease (ESKD) have a high symptom-burden and high rates of morbidity and mortality. Despite this, evidence has shown that this patient group does not have timely discussions to plan for deterioration and death, and at the end of life there are unmet palliative care needs. Advance care planning is a process that can help patients share their personal values and preferences for their future care and prepare for declining health. Earlier, more integrated and holistic advance care planning has the potential to improve access to care services, communication, and preparedness for future decision-making and changing circumstances. However, there are many barriers to successful implementation of advance care planning in this population. In this narrative review we discuss the current evidence for advance care planning in patients on dialysis, the data around the barriers to advance care planning implementation, and interventions that have been trialled. The review explores whether the concepts and approaches to advance care planning in this population need to be updated to encompass current and future care. It suggests that a shift from a problem-orientated approach to a goal-orientated approach may lead to better engagement, with more patient-centred and satisfying outcomes.

**Graphical abstract:**

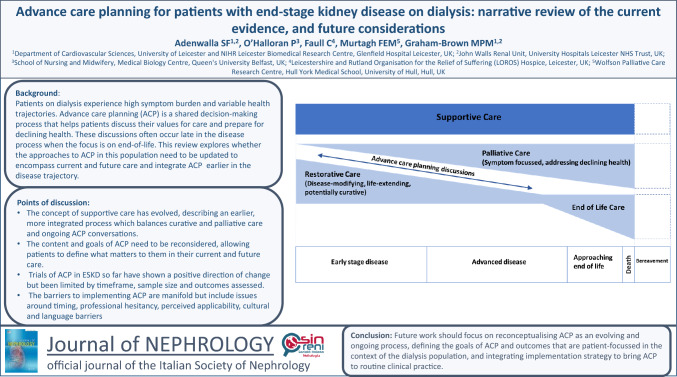

## Introduction

In the UK, treatment withdrawal is a rising mode of death in patients on dialysis, overtaking cardiovascular disease as the leading cause of death in patients over 65 years of age [1]. This suggests that the demographics of dialysis patients, and patient expectations from dialysis, may be changing in such a way that planning for deteriorating health and end-of-life may be becoming increasingly relevant.

For patients with end-stage kidney disease (ESKD) on dialysis there are a variety of unique challenges to having timely and effective discussions preparing for deterioration and dying. Patients with ESKD are at high risk of hospitalisation, intensive care admissions, and death. They are more likely to die in a hospital setting than at home or in a hospice [2]. This disparity is especially marked in comparison to those on a conservative care pathway, where those on dialysis have up to a 60% increased risk of emergency hospital admission and are more likely to die in hospital with little palliative care input [3]. Despite this, the proportion of people who have timely, effective discussions regarding resuscitation, withdrawal of dialysis and interventions aimed at maximising quality of life is low, and at the end of their lives there remain unmet palliative care needs [4]. There are also increased hospital costs associated with the last few years of life, irrespective of place of death or comorbidities [5].

In the international literature, advance care planning in the chronic disease population increases uptake of palliative services, improves symptom control and decreases anxiety for patients and families [6]. It is widely recommended for people with chronic illness and multi-morbidity, of which people on dialysis are one significant group. In ideal terms, advance care planning is a shared-decision making process that aims to support adults in understanding and sharing their personal values and preferences regarding future medical care during serious and chronic illness [7]. It encourages discussions with family and others, aiming to ease the transitions between treatment withdrawal, palliation, and preparation for their last days of life. Shared decision-making is a collaborative process that involves a person and their healthcare professional working together to reach a joint decision about care. Good practice for shared decision-making has been described by the National Institute for Health and Care Excellence (NICE) and it involves embedding a culture which enables such discussions at an organisational level, tailoring the methods used to support decision-making to the setting and context, offering carer or advocate support, and using high quality and accessible resources [8]. This should be the framework for integrating high quality and effective advance care planning into care for a population, however there are many barriers to successful implementation, partly because the practical definition and application of advance care planning varies widely.

The utilisation of advance care planning in dialysis populations is low [9, 10]. There is little evidence that advance care planning in this population influences discussions regarding preferences for care, subsequent impact on potentially avoidable hospital admissions, or the nature of the treatment required and delivered at the end of life [11]. The focus of care for this population does not always address the burdens of their advancing illness, which is crucial as the illness trajectory and symptom experience are highly variable. There is varied terminology used for the decision-making and care in advanced chronic kidney disease (CKD), which can cause confusion and contribute to stigma, affecting uptake of advance care planning processes. Additionally, there are cultural and socio-economic barriers to effective implementation of advance care planning that have been described and remain challenging to overcome [12].

In this article, we will review the current evidence for advance care planning in patients on dialysis and describe the challenges for consistent and timely implementation, including issues around the scope and goals of relevant advance care planning as well as the terminology that is used. We will also discuss ways advance care planning might be better implemented, and future areas of research.

### Search methodology

This is a narrative literature review so a comprehensive pre-determined search strategy was not specified. PubMed was used as the primary database to identify key papers using the search terms below:

(((((((("end stage kidney disease") OR (haemodialysis)) OR (dialysis)) OR ("peritoneal dialysis")) OR (hemodialysis)) OR ("end stage renal disease")) OR ("end stage renal failure")) OR ("chronic kidney disease")) AND ((((((((("advance care planning") OR ("supportive care planning")) OR ("advance care plan")) OR (advance directive*)) OR ("anticipatory care plan")) OR ("living will")) OR (DNACPR)) OR (DNAR)) OR ("end of life care plan")).

Forward and backward citation searching from key review articles and trial papers was also used.

## Terminology—language matters

Advance care planning is a shared decision-making process offered to patients with advanced illness or those approaching end of life. However, there are multiple other terms which describe overlapping concepts; advance care planning, end-of-life care planning, supportive care planning, and conservative care are all terms that have traditionally come under the auspices of palliative care approaches to chronic disease management. Awareness of these concepts amongst patients and clinicians in the general population is variable and terms are often used interchangeably [13, 14]. There is considerable heterogeneity with the way these terms are used in scientific literature which may reflect that pertinent elements occur as a continuum, but for the purposes of this discussion we have summarised these concepts in Table 1. The confusion and stigma around the use of these terms undoubtedly hinders the development of evidence, delivery of care and communication between patients and professionals [15, 16].

**Table 1:** A table summarising the terminology used when formal discussions are held with patients with end-stage kidney disease to help plan deterioration, symptom management and end of life

Term	Summary
Supportive Care	“Involves services that are aimed at improving the health-related quality of life for patients with established CKD, at any age, and can be provided together with therapies intended to prolong life, such as dialysis. Supportive care helps patients cope with living, as well as dying, regardless of life expectancy. Hospice/terminal care, also referred to as end-of-life care, shares the same philosophy, but it is under the larger umbrella of supportive care, and it is typically limited to patients who are believed to be within months of death” [24]
Advance Care Planning	“Advance care planning (ACP) enables individuals who have decisional capacity to identify their values, to reflect upon the meanings and consequences of serious illness scenarios, to define goals and preferences for future medical treatment and care, and to discuss these with family and health-care providers. ACP addresses individuals' concerns across the physical, psychological, social, and spiritual domains. It encourages individuals to identify a personal representative and to record and regularly review any preferences, so that their preferences can be taken into account should they, at some point, be unable to make their own decisions” [7]. It may include completion of documentation such as advance decisions, advance statements or forms such as the Recommended Summary Plan for Emergency Care and Treatment (ReSPECT) forms [68]. ReSPECT forms are widely used in the UK and aim to be “easy to recognise and [record] anticipatory recommendations about CPR and about other aspects of a person’s care or treatment (including but not limited to other life-sustaining treatment) if they suddenly become unwell and unable to make choices"
Advance statement	Allows general statements to be documented describing wishes and preferences for future care, should the person be unable to make or communicate preferences at that time. It can include aspects such as religious beliefs, social circumstances and food and drink preferences. These are not legally binding but can be taken into account by those making best-interests decisions on the person’s behalf [69]
Advance Decision to Refuse Treatment (also termed ‘Advance Directive’)	Whilst a person has mental capacity, they may document which medical treatments they would not want in certain circumstances, in the event that they do not have capacity to refuse at that time. It is time- and decision-specific and can be legally binding if certain criteria are met [69]
End-of-Life Care	“Supports those with advanced, progressive, incurable illness to live as well as possible until they die. It enables the care needs of both patient and family to be identified and met during the last phase of life and into bereavement. It includes management of pain and other symptoms and provision of psychosocial, spiritual and practical support” [18]
Comprehensive Conservative Kidney Care	“Holistic patient–centred care for patients with stage 5 [GFR category 5] CKD that includes: interventions to delay progression of kidney disease and minimize risk of adverse events or complications; shared decision making; active symptom management; detailed communication, including advance care planning; psychologic support; social and family support; cultural and spiritual domains of care. Comprehensive conservative care does not include dialysis” [70]

*ACP* advance care planning, *CKD* chronic kidney disease, *GFR* glomerular filtration rate

For many patients, some of these concepts have come to be associated with a phase of life when there are no options left for disease-modifying therapy and withdrawal of care is the main tenet. This means conversations with patients and referrals to palliative services may be delayed by professionals, and if seen to accept palliative care, patients may feel like they are giving up [17]. National guidelines in the UK encourage integration of advance care planning in kidney care, but often the focus is on planning for end-of-life care and the decisions needed for the last days of life [18]. In clinical practice, advance care planning conversations tend to aim for completion of documentation like advance directives or forms that focus on ceilings of treatment such as the Recommended Summary Plan for Emergency Care and Treatment (ReSPECT) forms. These documents are useful for any patient (not just those with chronic disease) whose prognosis is uncertain and where there is concern about imminent deterioration. However, such practice does not consider that a person, such as someone on dialysis, may live with an advanced condition for many years benefiting from therapy which aims to cure or slow disease progression whilst also suffering from the burden of disease and treatment side effects. It also does not clearly acknowledge that patients on dialysis have an unpredictable illness trajectory, where up to 50% of patients initiating dialysis over the age of 65 may die within the first year [19]. Whilst some of the discussion around the terminology used to describe conversations about death and dying may seem semantic, they are crucial to help destigmatise these conversations and allow them to be had in an appropriate and timely way to support people to live well, rather than to simply prepare for death. Indeed, for patients on dialysis, misunderstandings around frequently used end-of-life terminology are obstacles to having these conversations earlier in the patient journey when restorative care may still be a focus and impedes improvement of quality of life and end-of-life outcomes [20].

**Fig. 1:**
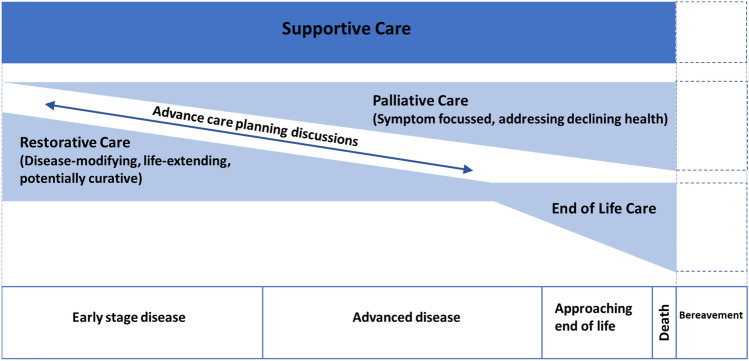
A figure demonstrating the over-arching concept of supportive care and the balance of restorative, palliative and end-of-life care at different stages of disease. Advance care planning discussions should ideally be started early and be an iterative process. Adapted from [23, 24]

Recognising that patient demographics and needs are evolving, the concept of supportive care has been developed as an overarching framework under which advance care planning is presented as an evolving discussion (Fig. 1). An important distinction from traditional definitions is that the sole focus is not on the dying process. Supportive care begins earlier, even prior to definitive diagnosis, describing a changing balance between restorative (curative) therapy and palliative care, encouraging integration of palliative care principles early in the disease trajectory. Helping patients and family adjust to a stepwise decline can give more time for complex discussions about future care and make changes to independence less abrupt. There is also evidence to suggest that this terminology is more acceptable to patients and healthcare professionals [21]; for one palliative oncology service, the change in clinic name to supportive care led to a 41% increase in referrals [22].

When services aim to integrate palliative care principles and expertise within routine clinical kidney care it can be termed ‘kidney supportive care’. It was recognised as a key requirement for patients with advanced CKD, and defined as distinct to conservative kidney care in a consensus document published by Kidney Disease Improving Global Outcomes (KDIGO) in 2015 [24]. Some countries such as the USA and Australia have started to establish supportive care practices within nephrology, but overall, practical implementation and translation into measurable outcomes has remained variable, and the service does not always include those on dialysis [11, 25]. In one survey in New Zealand, healthcare professionals described kidney supportive care as equivalent to usual care for CKD patients, with a smaller number viewing it as the same as conservative care or palliative care. Nevertheless, change in terminology had an impact; there was reported preference for referring patients with kidney disease to supportive care clinics over palliative services, for help with issues such as symptom management, complex treatment decisions, clinical or functional deterioration and requests to stop dialysis [21].

### The content of advance care planning discussions—the goals of care

The limitations and shifts in current terminology reflect that the content and goals of advance care planning conversations need to change. As described in this review, much of the current focus on advance care planning in policy, guidelines and research is on preparing for the time surrounding imminent death or about making decisions about the future or in the event of loss of capacity. The need to change the focus of advance care planning so that it can start earlier but remain relevant has been recognised in the wider advance care planning literature. Abel et al. framed it well by suggesting that the priorities of the discussion should be about ‘what matters most’ when patients are well or unwell, and how these priorities can be met with their support network at different health statuses [26]. This contrasts with the traditional definitions of advance care planning which concentrate on making decisions about avoiding or limiting treatments which patients may not have had cause to encounter when feeling well and stable. The Serious Illness Conversation Guide is an example of a resource that offers a structure for conducting advance care planning discussions that is more goal-concordant and incorporates a shared-decision making approach. The structure lends itself to helping patients and health care professionals evaluate present needs as well as introducing the concept of future care needs [27]. Pilot work in outpatient dialysis settings have shown that patients find the guide acceptable and worthwhile, and larger scale implementation is awaited [28, 29].

If advance care planning discussions were re-framed for patients around how to live well on dialysis, encompassing immediate and advance decisions at each life stage, it might lead to earlier engagement in advance care planning discussions. Earlier engagement may, in turn, improve access to restorative therapies and better prepare patients and families for later conversations, allowing re-evaluation of decision-making about death and dying towards the ends of their lives. This will not be possible without further work and consensus on defining the aims of advance care planning and measuring successful advance care planning in a productive way that is relevant to all stakeholders. The pathways and systems that address the issues raised in such advance care planning discussions would need to be established and need involvement from the wider multidisciplinary team to allow for an individualised approach that considers a patient’s specific biological, psychological, and social factors as well as cultural and religious beliefs.

## Engagement with advance care planning

The importance of routine, integrated advance care planning for chronic disease and multimorbid populations is recognised in policy and guidelines [7, 30]. Whilst receptivity towards advance care planning is largely positive, it is a very context specific intervention and this may be why uptake and measurable impact of advance care planning is variable and not consistently applied across settings and populations [31]. For patients with kidney disease, the last two decades have seen a concerted effort to raise awareness of advance care planning in the UK [18, 32]. However, the translation into improved outcomes at the end of life remains largely under-achieved. In 2011, a survey by McAdoo et al. found that of 94 patients on dialysis who died during an inpatient admission, only 28% had discussed end-of-life issues with their medical team prior to death [9]. Palliative care involvement, management of symptoms and withdrawal of dialysis prior to death were also low in the population surveyed, and particularly low in non-Caucasian patients. Similar findings have been replicated in more recent studies in the UK and other countries [10, 33–35]. In a survey of healthcare professionals in Australia and New Zealand, only 17% said they routinely discuss advance care planning with dialysis patients, with many citing barriers ranging from professional, environmental and cultural [35]. Preferences for care are more frequently discussed with family, with 50% of the population surveyed by Ladin et al. having discussed end of life with their social network, however given the high risk of mortality and morbidity for people on dialysis you might expect this number to be higher [20]. For those who do participate in advance care planning, discussions are often limited to ceiling of treatment or end-of-life scenarios, and decisions around dialysis seem to be decoupled from other aspects of advance care planning, such as cardiopulmonary resuscitation and ventilation which are discussed more frequently [33]. This may be due to a lack of discussion when initiating dialysis, leading to misconceptions about the limitations of dialysis and CPR, when dialysis cannot be used to sustain life, and what happens when dialysis is stopped. This might be because as dialysis becomes a part of everyday life, patients and professionals tend to focus on issues around comorbidities and dialysis adequacy rather than the overall trajectory [36].

## The evidence for advance care planning interventions: randomised controlled trials

A Cochrane review in 2016 highlighted that there is “sparse data” around the long-term outcomes of advance care planning in patients with ESKD [11]. Only two randomised controlled trials (RCTs) were included, and the authors felt that there was a lack of high-quality evidence and the concept of advance care planning was inconsistently defined. The review focussed on the outcomes of resuscitation measures and dialysis withdrawal, reflective of a more traditional, treatment-orientated approach. Considering the findings, the authors recommended that consistent methods are needed for reporting relevant and patient-centred measures. It was suggested that the lack of evidence reflected the variable real-world implementation of advance care planning in healthcare settings. A systematic realist review in 2018 explored this further and synthesised the trials in this population to identify implementation theories and explore the challenges to delivering interventions [37]. They found that successful interventions involved adequate training for healthcare professionals related to the intervention, and processes that can adapt to the individual’s social and cultural background, with possible surrogate inclusion where appropriate.

Details of major RCTs to date that have tested advance care planning in the ESKD population, characteristics of the interventions, and outcomes are summarised in Table 2. Most of the focus has been on planning for care in the period directly related to the end of life. Broadly, interventions have included peer mentoring [38, 39], written material [38], and trained facilitators leading discussions [39–42].

**Table 2:** A table summarising the randomised controlled trials that have explored advance care planning interventions in patients with end-stage kidney disease on haemodialysis

Author (year), country	Population	Intervention/comparator	Outcome(s)	Results
O'Halloran P (2020), Northern Ireland	Participants ≥ 65 years with ESKD on HD, identified by their nephrologist has having worsening symptoms, functional decline, ≥ 2 comorbidities and not expected to die in the next 3 months. Participants must be able to speak English. Surrogates also recruited	ACP discussions (baseline, 2 weeks and 12 weeks) with a trained nurse facilitator (with support from an ‘expert patient’) vs deferred entry. As part of the intervention, participants were offered the opportunity to complete a ‘Record of my wishes’ form	Quality of life, anxiety, depression, and wellbeing measures, perception of shared decision-making and agreement between patient and surrogate. Other outcomes were related to feasibility e.g. recruitment, retention and participation rates, acceptability of the intervention, estimated resource use and costs	Recruitment: recruitment lasted 189 days, with an intervention and data collection period of 443 days. Out of 67 patients invited, 30 declined. 36 patients were randomised (1 withdrew after consenting). Participation: of all 36 patients, 22 made an ACP. Only one patient chose to involve an expert patient. Surrogate involvement was low, with 17 patients able to identify a surrogate and 10 surrogates participating in the ACP process Retention: at 24 weeks, 17 (47%) patients remained in the trial Cost consequence: There was no statistically significant difference in health system resource use or costs between the immediate and deferred group for any cost category A statistically significant change in the effects, burden, and SF12 physical subscales of the quality-of-life measure was found
Song (2015), USA	Adults aged ≥ 18 years, identifying as African American or White, on HD or peritoneal dialysis for > 6 months, CCI ≥ 6 or CCI ≥ 5 with hospitalisation in the last 6 months. Participants must be able to speak English	A psychoeducational intervention consisting of 2 sessions with patient and surrogate, delivered by a trained nurse	Primary outcomes: preparation for end-of-life decision making; included dyad congruence on goals of care at end of life, patient decisional conflict, surrogate decision-making confidence, and a composite of congruence and surrogate decision-making confidence. Secondary outcomes: bereavement outcomes, included anxiety, depression, and post-traumatic distress symptoms completed by surrogates after patient death	210 dyads of dialysis patients and their surrogates. Dyad congruence and surrogate decision making were better in the intervention group than controls, but patient decisional conflict did not differ. Forty-five patients died during the study. Surrogates for patients in the intervention group had less distress-related symptoms than controls
Kirchhoff (2012), USA	Patients (and surrogates) with either end-stage heart failure or ESKD, at risk of serious complication or death in the next two years, receiving outpatient medical care. Did not specify language criteria or availability of translators	One ACP interview delivered by a trained facilitator vs usual care	Documented preferences were compared to care received at end of life (surrogate interviews and medical charts)	134/313 patients had ESKD. 110/313 died. Patients frequently continued to make their own decisions about care to the end (74%). The experimental group had fewer (1/62) cases where patients could not get their wishes met about CPR than control (6/48) (not a statistically significant difference). Significantly more experimental patients (38%) withdrew from dialysis than control (17%)
Song (2010), USA	Patients of African American heritage > 18 years, with ESKD receiving HD or PD ≥ 3 months (and their surrogates). Did not specify language criteria or availability of translators	One ACP interview delivered by a trained facilitator vs usual care	Feasibility and acceptability of ACP among African Americans with ESKD and their surrogate decision makers; to examine the preliminary effects of ACP on the following outcomes: (a) patient's level of difficulty in making choices, (b) patient–surrogate congruence in end-of-life care preferences, (c) surrogate’s level of comfort in decision making, and (d) psychospiritual well-being of patient and surrogate	19 patient/surrogate pairs—10 were randomised to the intervention, and 9 completed the intervention. There was greater congruence and in the intervention dyads (statistically significant). ACP was not shown to significantly decrease decisional conflict or increase surrogate decision-making confidence
Perry (2005), USA	Patients > 18 years with ESKD on dialysis. Participants must be able to speak English	(1) Peers were trained through advance directive workshop. Peers had eight contacts with patients to discuss ACP. (2) Printed material in a question and answer format. (3) Usual care	Completion of an advance directive; how comfortable the patient appeared to be in discussing advance directives	280 patients recruited. 22/63 (35%) patients in the peer intervention group completed an AD. 7/59 (12%) in the printed materials group and 8/81 (10%) in the control group. Patients in the peer-intervention group had a significantly greater level of comfort with AD discussion (mean score, 4.21 ± 1.18) than patients in groups 2 (mean score, 3.63 ± 1.23) or 3 (mean score, 3.63 ± 1.27; *F*2,196 = 4.67; *P* < 0.05)

*ACP* advance care planning, *ESKD* end-stage kidney disease, *CCI* Charlson Comorbidity Index, *CPR* cardiopulmonary resuscitation, *HD* haemodialysis, *PD* peritoneal dialysis

The effects of ‘advance directive workshops’ which include printed information and peer mentoring have been explored [38]. This study noted that peer mentoring was effective amongst patients of African American heritage (38% of the participants) in improving completion of advance directives and reducing distress, although the effects were less for White patients. This trial was not powered to detect differences by ethnicity so should be viewed as hypothesis-generating. The SPIRIT trials have iteratively tested a nurse-led model of facilitating end-of-life planning discussions between patients and their chosen surrogates, with a further, larger trial planned assessing effectiveness of the intervention [40, 41, 43]. Data showed that patient-surrogate congruence and decision-making was improved in the intervention group compared to control participants, although individual uncertainty about decisions did not differ between trial groups [40].

Early trials [38, 41, 42] tested advance care planning interventions as one-off interactions, however follow-up discussions to help support patients and surrogates through changing circumstances have been felt to be useful and more recent trials have incorporated them [39, 40]. A feasibility trial by O’Halloran tested nurse-led advance care planning discussions over a 12-week period in patients over the age of 65. The trial highlighted the current challenges to recruitment and resources in conducting advance care planning research in this population [39]. Issues included limited eligibility, recruitment, attrition and long trial duration. There was no difference in health system resource use between groups and the small sample size means that conclusions cannot be made about the effects of the intervention on advance care planning decisions. The authors recommended that future trials should approach patients earlier in their disease trajectory and recruitment should account for high rates of attrition. Whilst the intervention was broadly acceptable to most patients, there were issues of patient-surrogate understanding and the burden of research.

Trial evidence so far shows a positive direction of change, with good foundations testing the feasibility and acceptability of interventions. However, the issues outlined in the 2016 Cochrane review are still apparent and there is limited evidence on how advance care planning should be implemented in a real-world setting. There is still a lack of medium and long-term outcome data and it is unclear whether the goals and outcomes used in trials are relevant for this patient group. Trialled interventions thus far have mostly focussed on planning for end-of-life eventualities, making decisions in the event of loss of capacity, and completion of formalised documentation regarding ceilings of treatment. This mirrors the main focus of advance care planning discussions that occur in clinical settings and the policies and guidelines that underwrite them [18, 32]. Given that patients on dialysis have varied trajectories with different health priorities and goals, future interventions need a broader scope of practice, to make advance care planning relevant to a wider ‘phenotype’ of patient.

## Barriers to implementation of advance care planning for patients on dialysis

The barriers to implementation of advance care planning in this population are manifold. The optimal timing for introducing the concept of advance care planning will vary by individual patient circumstances and openness to discuss the future. It is important to emphasise that done correctly, early conversations have the potential to maintain hope and realistic expectations, rather than cause distress [36]. Models of health behaviours have been applied as a framework for improving engagement with advance care planning. A study involving older people highlighted that effective programs must be able to adapt to individual readiness to think about advance care planning [44]. In patients who do not feel ready to talk about declining health, action-orientated approaches (such as completion of documentation) would be unlikely to change perceptions and engagement. An advance care planning intervention that encompasses addressing attitudes towards illness and the future, the emotional, and cognitive elements of decision making may be more successful. Changing perceptions and improving engagement may be a more valuable outcome in the long-term for an individual patient who was in the ‘precontemplation’ or ‘contemplation’ stage of behaviour change than completion of an advance care planning document, but these outcomes are more difficult to measure in a standard interventional trial.

It is not clear who is best placed to have advance care planning discussions, in what setting the discussions should be held or even what the goals of advance care planning should be. Some clinicians only discuss advance care planning in the context of discussing options for renal replacement therapy and not broader goals of care [10]. There are also divergent definitions on what constitutes advance care planning discussions; whilst some clinicians define and prioritise advance care planning as completion of documentation, patients are more likely to view it as a more holistic discussion addressing goals of care, prognosis and disease trajectory [10]. Often, advance care planning is treated as a single event interaction, which is rarely revisited. However, the variable illness trajectory of patients with ESKD means that iterative discussions may be more helpful, accepting that priorities and preferences may change over time. The challenge lies in recognising a changing health status and addressing this. Using the ‘surprise’ question has been suggested as a trigger to review advance care planning (“Would you be surprised if this patient died in the next 12 months?”). This was tested in patients with non-dialysis dependent CKD and found to predict survival, with moderate reliability [45]. However, integration of this prompt into electronic health records found poor engagement and ultimately modest differences in care [46, 47]. This prompt would not include patients who may die from sudden events, an important consideration for a population at high risk of sudden cardiac death, suggesting that time-based triggers are required as well [48]. Furthermore, it is not clear whether the goals of advance care planning should focus on achieving life goals in the context of chronic illness and preparing for decision-making at a time of future illness, rather than making decisions ahead of time [49]. What is clear is that to integrate advance care planning into routine care and make it relevant for this population, advance care planning goals and outcomes must be defined with patients.

Some healthcare professionals have reported hesitation in initiating advance care planning discussions without prompt from the patient due to a belief that they do not want the information, or concern about being unable to give a certain prognosis [50]. Often, discussions are only initiated around the time of crisis (and felt to be too late), even though health care professionals have noticed preceding deterioration but felt flagging this to the patient would be too distressing [50]. These perceptions are at odds with what is reported by patients with ESKD. Qualitative studies have demonstrated that patients expect doctors to raise the topic, some have expressed a preference that discussions are with their nephrologist, and accept that prognostic information may not be accurate [51]. Seeing fellow patients on a dialysis shift deteriorate and pass away can be a constant reminder of the tightrope of life and death of being on maintenance haemodialysis. Some feel unable to raise these fears for fear of burdening family or being pushed away by healthcare professionals [52]. Allied health professionals most often see themselves as a support system rather than responsible for raising advance care planning [53]. The ideal model is far from clear, but certainly consistency of communication and consensus on where responsibility lies are needed.

A repeated issue is the lack of time, training, and resources for professionals to take ownership over these discussions [37]. A study looking at barriers to implementation of an advance care planning intervention found difficulties in scheduling advance care planning sessions due to clinician availability, finding an appropriate space for discussions and challenges to meeting patients on non-dialysis days [54]. A scoping review exploring participant views on randomised trial participation (in general) found that most participants on haemodialysis preferred trial visits to take place either whilst receiving dialysis or around the dialysis visit, whilst others felt they may be too unwell on dialysis days [55]. However, this may be impractical for an advance care planning intervention, where it would be difficult to accommodate the need for privacy or presence of caregivers. Furthermore, global cognitive function has been shown to vary during the dialysis cycle; an important consideration when trying to optimise communication and patient capacity [56]. Advance care planning needs to be appropriately resourced, but this highlights the need for an ‘integrated knowledge translation approach’ when planning work embedding advance care planning into usual care, which means involving patients and stakeholders from project conception to dissemination of plans [57]. This approach aims to improve the relevancy and usefulness of research, with the potential to reduce research waste and improve uptake of interventions.

It has been consistently shown that fewer non-Caucasians participate in advance care planning discussions, palliative pathways and end-of-life planning [58, 59]. These differences can lead to inequitable access to end-of-life care services, such as hospice care and other specialist palliative services [60]. Of the randomised trials of advance care planning interventions in dialysis populations, only a few have included people of African American heritage, whilst people from Asian ethnic backgrounds have been largely unrepresented [38, 39, 41] (Table 2). There do appear to be differences around willingness to engage with end-of-life care and treatment withdrawal [41, 61, 62]. In a study by Ahn et al., the majority of African American patients on haemodialysis did not have an advance care planning, and it is possible that perceived cultural and language barriers dissuade healthcare professionals from broaching advance care planning discussions [62]. Those patients that did have end-of-life discussions with professionals and family were less likely to prefer aggressive life-extending care, suggesting that decision-making can be influenced by time to reflect and addressing perceptions. It must also be accepted that advance care planning interventions may help patients to choose options contrary to what healthcare professionals may recommend. In a pilot study of 19 patients, there was a trend for patients randomised to the advance care planning intervention to change their preferences towards life-sustaining treatment; the authors speculated that this could be the effect of the intervention challenging their individual decision-making processes, leading to choices that represent the path of least resistance. The other speculation was due to the influence of strong spiritual beliefs; for some, feeling that God decides the amount of ‘suffering’ that they can endure may affect their tolerance for aggressive treatment [41]. Exploratory analysis of the SPIRIT trial identified that the intervention had greater effect on some outcomes depending on ethnic group, highlighting that robust interventions need to be able to adapt to individual and demographic needs [61]. Furthermore, much of the qualitative work around patient experience of end-of-life care in ESKD has been limited to English-speaking patients [63]. The presence of a language barrier has been an exclusion criterion in many randomised controlled trials for pragmatic reasons (Table 2), and this is a clear limitation of the evidence base and the generalisability of study findings where language was a barrier to participation.

A multicentre qualitative study in the UK focussing on South Asian patients, receiving end-of-life care either still on dialysis or receiving conservative care, conducted interviews in the participant’s preferred language. The findings acknowledged the additional time that non-English speaking patients may need to have discussions; resources are needed to organise translators, conduct the consultation and possibly increase the frequency of appointments to overcome communication barriers [64]. The reliance on family members as informal translators may also introduce bias in communication. The difficulty of doing these things well in a crisis emphasises the importance of initiating advance care planning discussions at an early stage. Study participants found iterative discussions over time to be more effective, as this allowed a rapport to be developed and patients and families to come to terms with their prognosis. Furthermore, there can be an assumption that patients from certain backgrounds may not wish to take part in decision-making, but this needs to be identified on an individual patient basis. Future studies must make a concerted effort to represent multi-ethnic dialysis populations to try and build advance care planning interventions that are culturally sensitive.

## Future directions

The qualitative data around advance care planning in the dialysis population are irrefutable; more information about advance care planning is desired by patients and health care professionals. Observational and initial trial data suggest effective advance care planning has the potential to improve patient experience and care. A cost-effectiveness model of advance care planning in a dialysis population performed with Australian data suggests that advance care planning may be cost-effective, with an incremental cost-effectiveness ratio of AUD$28,421, but there are few real-world data [65]. Pockets of excellence for kidney supportive care practices exist [25], but the scope of practice can be variable; clinics do not always include dialysis patients and some focus on patients with ESKD on a conservative pathway.

Implementation of successful, consistent and integrated advance care planning aimed at the dialysis population will be a major challenge. Implementation science offers tools and strategies to integrate an intervention into complex settings, to promote sustainability of the approach, and increase impact and engagement. The frameworks emphasise the importance of considering the wider systems contexts as well as local and individual influences when introducing or scaling up an intervention. Implementation strategies have been explored in recent advance care planning studies [39, 54] but further integration of these principles into clinical trials are needed to bridge the gap between evidence and clinical practice: the ‘know-do’ gap [57]. A protocol for a study using implementation theory to demonstrate scalability of a discussion-led advance care planning intervention across multiple sites has been published, using implementation and evaluation-based outcomes to assess success for both patients and providers [66]. The 15-year experience of a kidney care network in Canada describing efforts to integrate palliative care principles into routine care for patients with CKD stage 4–5 could be used as an exemplar for other centres and to highlight the ongoing challenges [67]. Key learning points from their experience include the importance of considering network structure and organisation to enable cultural changes, the time required for such changes to become embedded, and the adaptability of strategies to local context. The authors felt that broader education for existing health care professionals allowed for utilisation of existing skill sets and had success with empowering local leaders to engage stakeholders. To aid implementation, trials have suggested that a successful and feasible advance care planning intervention should have outcomes that are process-related and patient-centred to reduce burden. Patient-related outcomes in this area are challenging to define and quantify and additionally, are intrinsically linked to what the goals of advance care planning should be. For example, there is a strong argument that we should not be solely focussing on asking patients to make decisions ahead of time but preparing them to have important conversations at a time of illness [49]. The 2015 SPIRIT trial measured ‘preparedness for decision-making’ through other surrogate outcomes such as patient-carer congruence and decision-making confidence [40]. A study that can link patient-related outcome measures to the decisions made and treatments delivered at the end of life would be useful to demonstrate the potential benefits of advance care planning to multiple stakeholders, ranging from patients to commissioners, but clearly much work is needed to define and refine many of these parameters.

At present, the focus of advance care planning in this population is too narrow, and largely centres around decision-making for specific medical decisions. To become more relevant and attract better engagement, advance care planning processes need to become more iterative, with a broader focus and with goals of care and outcomes that are defined by patients and stakeholders.

## Conclusions

It is accepted that there is need for earlier, more transparent, normalising of advance care planning discussions and priorities. There is still no consensus regarding ‘who, where, when and what’ needs to be considered when adapting advance care planning for this specific patient group. Trials thus far have been limited by time frame, size, population, and outcomes assessed. Future work should focus on reconceptualising advance care planning as an evolving and ongoing process, defining the goals of advance care planning and outcomes that are patient-focussed in the context of the dialysis population, and integrating implementation strategy to bring advance care planning to routine clinical practice.

## Data Availability

Data available on request.
